# Comparative Evaluation of the Effect of Custom-Made Anatomic Healing Abutment on Papilla Morphology and Gingival Aesthetics With That of Conventional Type of Healing Abutment: A Randomized Controlled Trial

**DOI:** 10.7759/cureus.101909

**Published:** 2026-01-20

**Authors:** Deepthi V Surendran, Rajagopal Ravichandran, Harshakumar Karunakaran

**Affiliations:** 1 Prosthodontics, Government Dental College, Thiruvananthapuram, Thiruvananthapuram, IND

**Keywords:** healing abutment, implant aesthetics, papilla index, pink aesthetic score, prosthodontics

## Abstract

Background

Gingival aesthetics has become inevitable in the overall success of implant-supported restorations. The interdental papilla plays a crucial role in attaining optimal aesthetics during the restoration of anterior edentulous regions with implants. The use of customized healing abutments helps to shape and condition the gingival tissue during the healing phase, thereby preserving its natural contours for the subsequent prosthetic stage.

Objectives

The primary objective is to compare the papillary morphology adjacent to implant restorations using the papilla index after the use of custom-made and conventional healing abutments at permanent crown insertion and at six months. The secondary objective is to compare the emergence profile aesthetics using the pink esthetic score (PES) at the same time points for both types of healing abutments.

Methodology

The study was designed as a randomized parallel group trial with an allocation ratio of 1:1. Patients who visited the Prosthodontic Department of Government Dental College, Thiruvananthapuram, for implant-retained restoration in the maxillary central incisor region were recruited for the study. Implants were placed in a delayed loading protocol. A total of 20 participants were selected for the study. The control group (n=10) included patients treated with conventional healing abutments, while the test group (n=10) consisted of patients who received customized healing abutments. After six months of healing, conventional healing abutments were placed at second-stage surgery for group I, whereas customized healing abutments were used for group II. Outcome variables, papilla index scores (PIS), and PES were evaluated and compared at the time of insertion of permanent restoration and after six months.

Results

The distribution of mean papilla index between group I and group II at insertion showed that there is a statistically significant improvement in both mesial and distal PIS for group II (2.00±0.00) when compared to group I (1.40±0.52). PES were compared between the two groups at the time of insertion of the final crown. The mean PES for the customized group was (11.40±0.52) while that of the conventional group was only (10.40±0.52) (P value <0.05). The PES of Group I and Group II were compared after six months of crown insertion. Mean PES for the customized group (12.40±0.52) when compared to the conventional group (11.40±0.52) was higher, and the difference was statistically significant. (P value <0.05) .Statistical analysis was done using Mann-Whitney U test.

Interpretation and conclusion

This study concludes that customized healing abutments help in procuring a better emergence profile and aesthetics of the restoration. It also restores the optimum contour and papillary size at a faster rate than the conventional healing abutment.

Clinical significance

The study is unique in that it compares the effect of customized healing abutment on gingival aesthetics and papillary morphology with that of conventional healing abutment in a delayed implant loading protocol. In delayed loading protocols, customized healing abutments are critical tools in guiding the healing of peri-implant soft tissues. They provide superior aesthetic outcomes, especially in the anterior aesthetic zone, by promoting natural-looking gingival architecture.

## Introduction

In recent years, there has been a significant rise in aesthetic demands within dentistry. Achieving visual and functional harmony of the soft tissues surrounding dental implants is critical, especially in the anterior aesthetic zone, where appearance is as important as function. The use of customized healing abutments is supposed to prepare the adjacent gingival tissue for the prosthetic stage [1,2]. Loss of papilla adjacent to the implant leads to an esthetic handicap [3]. In 1989, Albrektsson’s criteria for implant success were expanded to include the requirement that the implant must permit the placement of a restoration with satisfactory aesthetic appearance [4,5]. Thus, gingival aesthetics has become inevitable in the overall success of implant-supported restorations [6]. It is very difficult and challenging to recreate the soft tissue scaffold, which is necessary to achieve the illusion of natural teeth [7]. The presence of interdental papillae is critical in achieving optimum aesthetics in the restoration of the anterior edentulous area with implants [8]. Numerous surgical and prosthetic methods have been suggested by various clinicians for the preservation and recreation of interdental papilla [9-12]. But in this era of evidence-based dentistry, data-oriented scientific support is also inevitable for the acceptance of any such methods. A healing abutment is a prosthetic component attached to the exposed end of the dental implant used to shape the peri-implant soft tissues. Conventional healing abutments have circular cross sections, and hence an optimal shape of gingiva cannot always be achieved with conventional abutments [13]. However, custom “tooth form” healing abutments closely resemble the cross-sectional anatomy of the lost tooth. Many studies reported that custom healing abutments can be used to enhance the aesthetic outcome of implant therapy [14-16]. Hu et al. [17] studied the impact of healing abutments in healing of peri-implant tissue in immediate implant placement. However, there is a dearth of research on the effect of customized healing abutments on the final aesthetic outcome in delayed implant placement protocols.

The present study aims to establish whether there is an increase in interproximal papillary levels in cases with customized healing abutments compared with conventional healing abutments. It also focuses on finding out the effect of customized healing abutment on the final aesthetic outcome of the implant restorations.

Objectives

The primary objective is to compare the papillary morphology adjacent to implant restorations using the Jemt papilla index [18] after the use of custom-made and conventional healing abutments, assessed at permanent crown insertion and at six months. The secondary objective is to compare the emergence profile aesthetics using the pink esthetic score (PES) at permanent crown insertion and at six months for both types of healing abutments [19].

## Materials and methods

The study was designed as a randomized parallel group trial with an allocation ratio of 1:1 and was started after getting approval from the Institutional Ethics Committee, Government Dental College, Thiruvananthapuram (IEC/C/81A/2011/DCT dated 10/3/2011). The trial was registered in the Clinical Trial Registry of India (CTRI/2011/04/001671). The duration of the study was one year after getting the approval. The study started on 10/3/2011 and was completed on 10/3/2012.

Inclusion and exclusion criteria

A total of 35 patients who visited the Prosthodontic Department of Government Dental College, Thiruvananthapuram, for implant-retained fixed restoration in the maxillary central incisor region during this time period were assessed for eligibility for the study. Fifteen patients were excluded, as nine did not meet the inclusion criteria and six were unwilling to participate. Patients aged 18 to 35 years who had lost a maxillary central incisor due to caries or trauma were included in the study, provided they exhibited good oral hygiene based on the Simplified Oral Hygiene Index by Green and Vermillion [20], which evaluates both plaque and calculus levels. Patients were included only if they consented to the delayed implant loading protocol and exhibited no midline diastema, adequate attached gingiva, and sufficient bone volume at the implant site (faciopalatal width >5.5 mm, mesiodistal space >6.5 mm, and bone height >12 mm). Patients were excluded if they exhibited insufficient bone quantity at the proposed implant site, severe intermaxillary skeletal discrepancies, or parafunctional habits such as bruxism. Individuals with a history of smoking, alcoholism, or drug abuse, as well as pregnant or lactating women, were excluded. Patients who had undergone treatment for malignancy, those with systemic disorders, or those receiving long-term steroid therapy were also exempted. A total of 20 participants were recruited for the study.

Study groups and surgical protocol

Participants were randomly allocated into two groups based on the type of healing abutment used in the second-stage surgery. *Group I (Control Group)* After three months of healing, conventional healing abutments were placed in the second stage of surgery (n=10).

Group II (Test Group)

After three months of healing, customized healing abutments were placed in the second stage of surgery (n=10).

**Figure 1:**
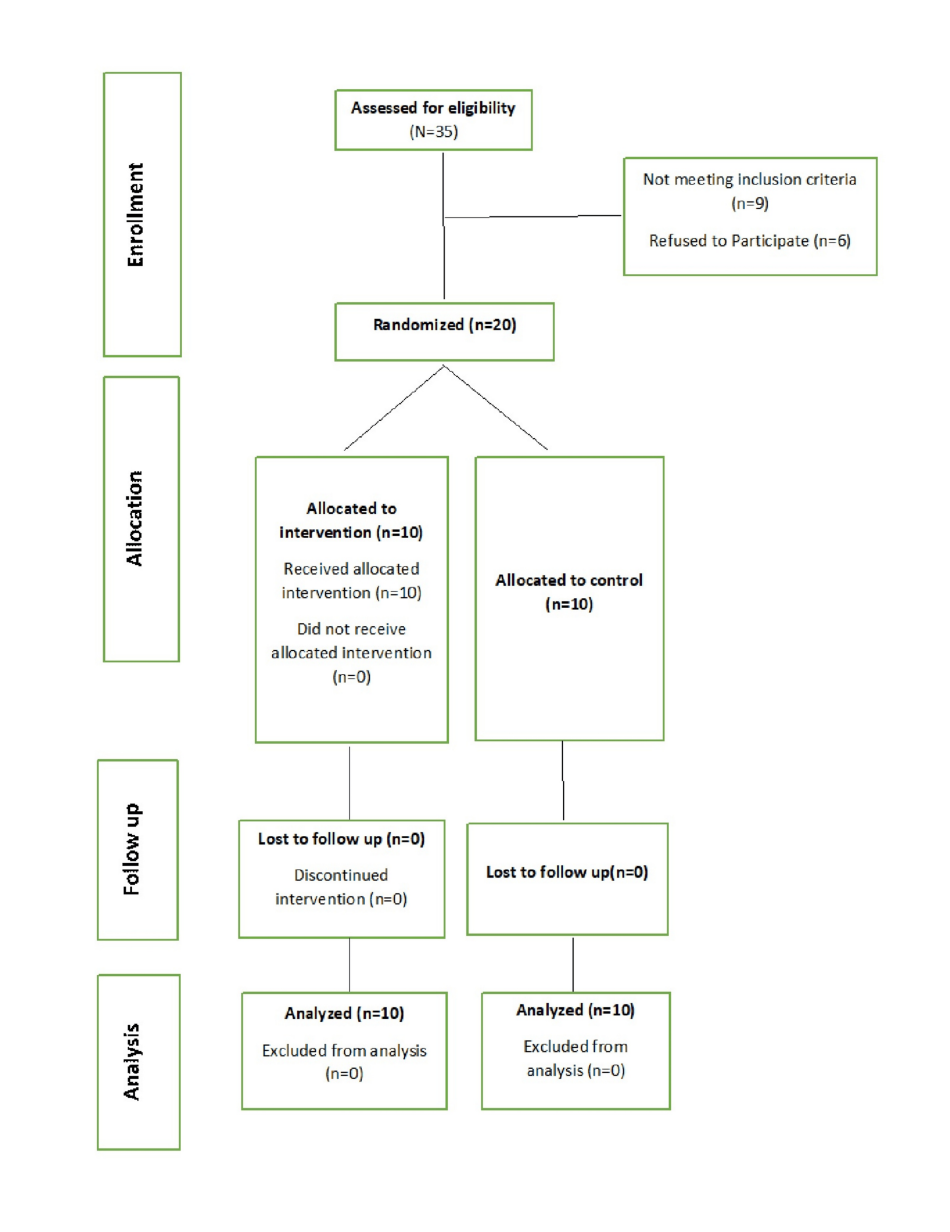
Consort flow diagram

Randomization was carried out using a computer-generated random sequence. Allocation concealment was ensured by using sealed, opaque, sequentially numbered envelopes that were opened only at the time of intervention. The study followed a triple-blind design, in which the participants, the outcome assessors, and the statistician were blinded to the group allocation to minimize bias. The study was conducted in accordance with the Helsinki Declaration. Informed consent was obtained from all study participants. The CONSORT flow chart of the study is given in Figure 1.

Sample size

The sample size was estimated using G*Power version 3.1. The study was powered at 80% (β=0.20) with a significance level of 5% (α=0.05). Based on pilot data (n=5 per group) showing mean values of 2.0 and 1.4 (difference Δ=0.6) with a pooled standard deviation of approximately 0.37, a minimum of six participants per group was required to detect the specified difference. To account for potential dropouts, the sample size was increased to ten participants per group.

Preoperative assessment

Necessary blood investigations, including coagulation profile, routine blood counts, fasting blood sugar, and viral screening, were performed to assess the health status of each patient. Available bone was evaluated for height, width, uniformity, and presence or absence of any pathology using an intra-oral periapical radiograph and panoramic radiograph. Bone mapping was done to evaluate the soft tissue thickness, the bone width, and contour. 

Intervention

All patients received a single oral dose of 2 grams of amoxicillin (or 600 mg of Clindamycin in cases of Amoxicillin allergy) one hour before implant surgery. After draping, the extra-oral tissues were disinfected with Betadine solution. For intra-oral disinfection, patients were given 1.2% Chlorhexidine mouthwash just before the procedure. Local infiltration or regional nerve block with 2% lignocaine with epinephrine was given as local anesthesia. A mid-crestal incision without vertical releases was given for flap reflection. A full-thickness mucoperiosteal flap was elevated in all selected patients. Osteotomy preparations were done using sequential drilling under copious saline irrigation. A recommended speed of 1200 rpm was selected for all the drills. Complete seating of the Adin Dental Implant System (Adin Dental Implant Systems Ltd, Afula, Israel) implant was ensured by close approximation of the crest module of the implant to the crestal bone.

The implant diameter was selected such that there was a minimum of 1.5 mm space mesio-distally between the implant and the adjacent natural teeth. The implants were placed by the same operator to eliminate operator variability. Care was taken to preserve a minimum of one mm of buccal and lingual cortical bone, and the crest module was kept flush with the crestal bone. In Group II patients, where customized healing abutments were planned, an open tray impression coping was connected to the implant, and a bone level impression of the fixture was taken in light body elastomeric impression material (Elite HD, Zhermack, Italy) using a custom tray, which was disinfected using Glutaraldehyde 2% solution (Cidex ASP India Pvt. Ltd., Mumbai, India). 

**Figure 2:**
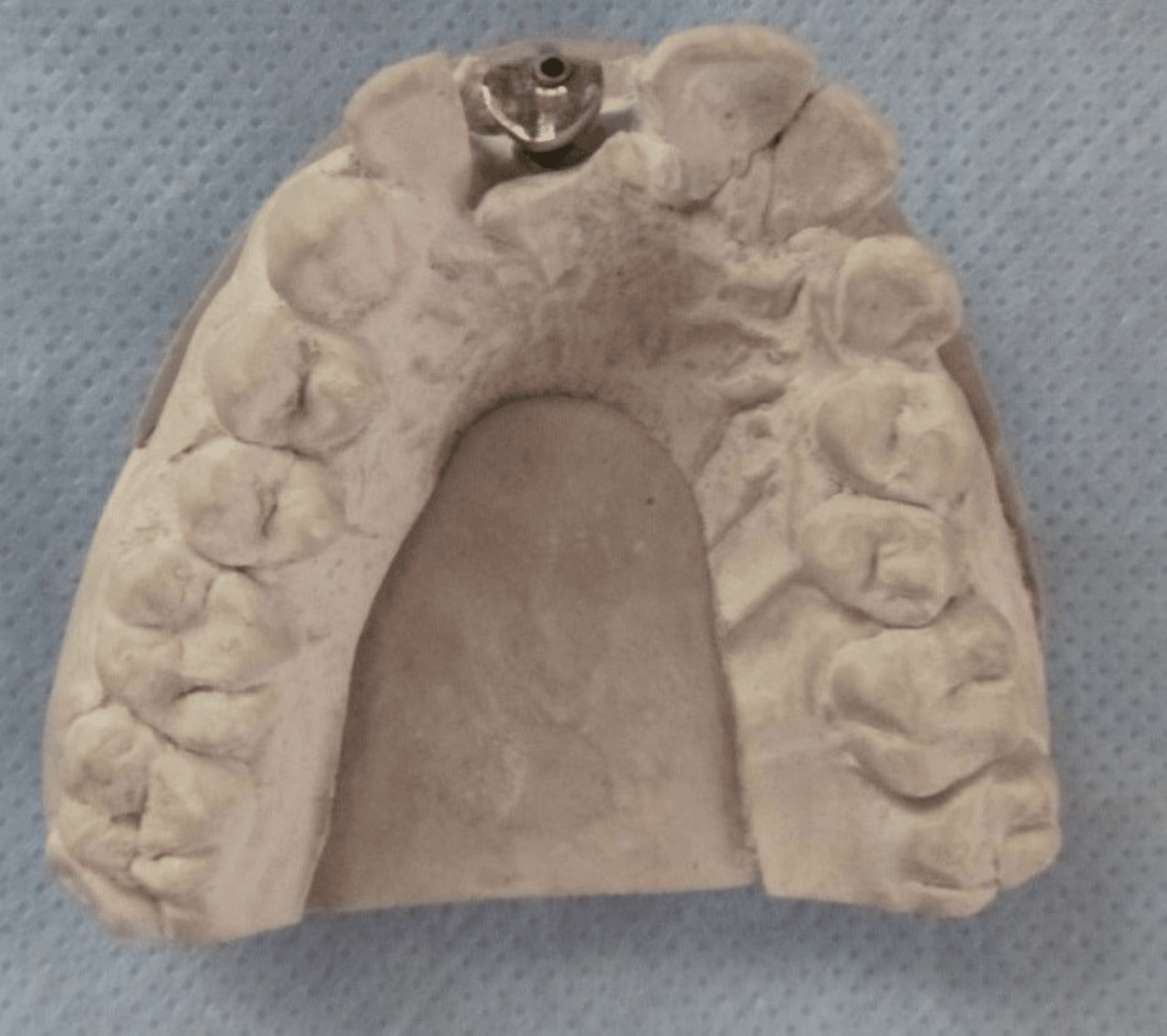
Customized healing abutment

In Group II (Test group), a plastic abutment was attached to the implant analog of the cast. Then, this abutment was modified in shape using inlay wax simulating the contralateral incisor at a gingival level cross-section. This wax pattern was later cast in Cp Titanium grade 4 American Society for Testing and Materials (ASTM) F67 (Orotig S.p.A., Verona, Italy) (Figure 2).

In case of uneventful healing, the patients were recalled after three months. The implant was exposed by a small semilunar incision. The cover screw was replaced with conventional healing abutments in group I and customized healing abutments in group II patients.

The patients were recalled after three weeks, and healing abutments were removed in order to place the impression coping attached to the fixture. Impressions were taken using Polyvinyl Siloxane impression material (Elite HD, Zhermack, Italy), consistency using a custom tray, and open tray impression coping. In group II, the impression post was modified using composite in order to support the newly formed gingival cuff after three weeks. In all cases, porcelain-fused-to-metal restorations were given.

Outcome variables

The evaluation phase was conducted in the Department of Periodontology, Government Dental College, Thiruvananthapuram. The evaluators were blinded in the study.

Phase I - at the time of crown insertion, and Phase II - after six months of crown insertion. During the evaluation of each phase, two parameters were checked and recorded. 1) PIS by Jemt; 2) PES by Rudolph Furhauser [19].

PIS by Jemt [18].

Swedish researcher Dr. Torsten Jemt (1997) created a five-point index to assess the size of the interproximal gingival papillae adjacent to single-tooth implants: Score 0 shows no papilla and no indication of curvature of the soft tissue adjacent to the implant crown. Score 1 shows less than one-half of the papilla height is present, and a convex curvature of the soft tissue adjacent to the implant crown and adjacent tooth can be observed. Score 2 shows at least one-half of the papilla height but not up to the contact point between the teeth. The papilla is not completely harmonious with the adjacent papillae between the permanent teeth. Soft tissue is harmonious with adjacent teeth. Score 3 shows the papilla fills the entire proximal space and is harmonious with the adjacent papillae. Here, the soft-tissue contour is optimum. Score 4 shows hyperplastic papillae and papillae covering the implant or the adjacent tooth surface in excess. Here, the soft-tissue contour is found to be irregular.

PES

PES is based on seven variables: mesial papilla, distal papilla, soft-tissue level, soft-tissue contour, alveolar process deficiency, soft-tissue colour, and texture. Each variable was assessed with a 2-1-0 score, with 2 being the best and 0 being the poorest score. All other variables were assessed by comparison with a reference tooth, which is the corresponding tooth in the arch. The highest possible score showing an exact match with the reference tooth was 14 (Table 1).

**Table 1:** Pink esthetic score The pink esthetic score (PES) was developed by Furhauser et al. to evaluate the esthetic outcome of soft tissue around implant-supported single crowns in the anterior zone [19].

Variables	0	1	2
Mesial papilla	Absent	Partially fills the place	Completely fills the place
Distal papilla	Absent	Partially fills the place	Completely fills the place
Level of soft-tissue margin	Major discrepancy >2 mm	Minor discrepancy 1 to 2 mm	No discrepancy <1 mm
Soft-tissue contour	Unnatural	Fairly natural	Natural
Alveolar process	Obvious	Slight	None
Soft-tissue colour	Obvious difference	Mild difference	No difference
Soft-tissue texture	Obvious difference	Mild difference	No difference

Outcome analysis

Outcome analysis was done at the time of permanent crown insertion and six months after crown insertion. The Mann-Whitney U test was used to compare the mean papilla index values and PES of group I and group II, as the data were not normally distributed. Statistical analysis was performed using IBM SPSS Statistics for Windows, Version 25.0 (IBM Corp., Armonk, NY, USA). A P value of <0.05 was considered statistically significant.

## Results

There were no dropouts during the study period. All participants were followed up to six months. No adverse effects were reported.

An intergroup comparison between group I and group II at the time of crown insertion and after six months of insertion of the crown was done. Intra-group comparison of the soft tissue parameters at crown insertion and after six months of crown insertion for patients in group I as well as group II.

The distribution of mean papilla index between group I and group II at crown insertion showed that there is a statistically significant improvement in both mesial and distal PIS for group II (2.00±0.00) when compared to group I (1.40±0.52). The Mann-Whitney U test was used to do the statistical analysis for this comparison (Table 2).

**Table 2:** Comparison of PIS between group I and group II at insertion A P value <0.05 was considered statistically significant. PIS: papilla index score.

PIS (At Insertion)	Group	Median(IQR)	Mann-Whitney U Value	P value
Mesial	Group I (Conventional) (n=10)	1.00 (1)	20.000	0.023
	Group II (Custom) (n=10)	2.00 (0)		
Distal	Group I (Conventional (n=10)	1.00 (1)	16.000	0.008
	Group II (Custom) (n=10)	2.00 (0)		

PES were compared between the two groups at the time of insertion of the final crown, and statistical analysis was done using Mann-Whitney U test. The mean PES for the customized group was 11.40±0.52, while that of the conventional group was only10.40±0.52. As per this analysis, there is a significant improvement in PES for group II as compared to group I at the time of final crown insertion (P value <0.05) (Table 3).

**Table 3:** Comparison of PES between group I and group II at insertion A P value <0.05 was considered statistically significant. PES: pink esthetic score.

PES (At Insertion)	Group n=10	Median (IQR)	Mann-Whitney U Value	P value
Total	Group I (n=10)	10.00 (1)	12.000	0.004
	Group II (n=10)	11.00 (1)		

PIS were compared between the two groups after six months of final crown insertion, as mentioned. There was no significant change in the papillary size between the customized group (2.60±0.52) and the conventional group (2.20±0.42) at the end of six months (Table 4).

**Table 4:** Comparison of PIS between group I and group II after 6 months P value >0.05 was considered not statistically significant. PIS: papilla index score.

PIS (After 6 Months)	Group	Median (IQR)	Mann-Whitney U Value	P value
Mesial	Group I (n=10)	2.00(0)	30.000	0.07
	Group II (n=10)	3.00(1)		
Distal	Group I (n=10)	2.00(0)	30.000	0.07
	Group II (n=10)	3.00(1)		

The PES of Group I and Group II were compared after six months of crown insertion. Mean PES for the customized group (12.40±0.52) when compared to the conventional group (11.40±0.52) was higher, and the difference was statistically significant (P value <0.05) (Table 5).

**Table 5:** Comparison of PES between group I and group II after 6 months A P value <0.05 was considered statistically significant. PES: pink esthetic score.

PES (After 6 Months)	Group	Median (IQR)	Mann-Whitney U Value	P value
Total	Group I (Conventional ) (n=10)	11.00 (1)	26.000	0.03
	Group I (Custom) (n=10)	12.00 (0)		

Intra-group comparison

The same groups were compared at different times (at the time of insertion and six months) to find out whether there was any significant change in parameters over time.

A comparison of the PIS at the time of final crown insertion and after six months was done using the Mann-Whitney test. It was found that there was a statistically significant increase in the mean Papilla Scores both mesially and distally for both groups. Mesial and distal papilla scores increased from 1.40±0.52 to 2.20 ± 0.42 for the conventional group and the customized group from 2.00±0.0 to 2.60±0.52 mesially and from 2.20±0.42 to 2.60±0.52 distally. So it was inferred that there was improvement in papillary size and contour irrespective of the group with time during the observation period. For the conventional group, mean PES improved from 10.40±0.52 to 11.40±0.52, but the difference was not statistically significant. Mean facial mucosa level scores showed a decrease from 1.40±0.52 to 1.20 ±0.52. This observation may be due to isolated cases of recession of facial mucosa that were noticed after six months in the conventional group.

PES was measured for the custom group at the time of final crown insertion and after six months, and even though there was a slight improvement in the mean PES in the custom-made healing abutment group (from 11.40±0.52 to12.40±0.52), it was found to be statistically insignificant.

## Discussion

Implant replacement in the anterior aesthetic zone is one of the greatest challenges in implant dentistry [21,22]. The anterior maxilla often shows poor bone quality, which further complicates the condition. Hence, a conventional two-stage protocol becomes a feasible option in many of the cases. Even though this multistage approach guarantees long-term success in terms of function, but to recreate the lost interproximal papilla remains a challenge. According to Harzeler et al., soft tissue management can be done at four different stages, namely: before implant placement, at the time of placing the implants or during the healing phase of the implants, at the second stage of surgery, and in the maintenance phase [23]. Prosthetic-guided soft tissue healing means the early introduction of prosthetic components like custom-made healing abutments or implant-supported provisional restorations that correspond to the anatomy of the lost tooth to enhance the aesthetic outcome [24]. Intra-group comparison at the time of insertion and after six months of both groups shows significant improvement in the papillary size and contour during the six-month observation period. This indicates spontaneous soft tissue Intra-group comparison at the time of insertion and after regeneration in both groups in the early periods of healing. This finding is again supported by Priest [25] and Choquet et al. [26], who stated that papilla regeneration around the single implant is always a predictable outcome.

Chokree et al. evaluated customized versus prefabricated healing abutments in immediate implant sites and found that the customized group exhibited less change in PES after six months, findings consistent with the present study [27]. Nowadays, many polymer-based custom healing abutments are commercially available. A polymer named ployetheroether ketone (PEEK) is extensively used to make anatomic healing abutments. Abrahamsson et al. [28] documented that the material used in the abutment portion of the implant affects the quality of the attachment that occurs between the mucosa and the implant. In this study, custom-made healing abutments were cast in the same material as conventional healing abutments, which is titanium, to avoid the effect of abutment material variability in the outcome of the study. Accurate transfer of the healed tissue to the laboratory is equally important for the fabrication of a precisely fitting permanent restoration with a good emergence profile. Hence, impression coping should have the same gingival dimensions as the healing abutment so that there is no gap exists between the impression coping and the walls of the shaped gingival cuff. The papilla index used for evaluating the effect of custom-made healing abutments on the interproximal papilla takes into account not only the height but also the contour of the existing papilla. Intra-group comparison at the time of insertion and after six months of both the custom-made anatomic healing abutment group and the conventional healing abutment group shows that there is significant improvement in the papillary size and contour during the six-month observation period. This indicates spontaneous soft tissue regeneration in both groups in the early periods of healing. Hence, this study shows that customized healing abutments help in procuring a better emergence profile and aesthetics of the restoration. It also restores the optimum papillary size and contour at a faster rate than the conventional healing abutment.

Limitations and future scope of the study

However, further multicentre studies with larger sample sizes and extended follow-up periods are needed to validate and strengthen these findings. The present study focused exclusively on cases with a missing maxillary central incisor to maintain uniformity in anatomical characteristics and reduce variability between the groups. While this approach enhances internal validity, it also limits the generalizability of the results. Future research that includes a wider range of clinical scenarios, such as different tooth types, varied ridge conditions, and diverse patient populations, conducted across multiple centres, will provide more comprehensive evidence and allow the results to be extrapolated more reliably to the general population. In this study, the follow-up period was limited to six months. The lack of long-term follow-up represents a limitation, as it remains unclear whether the initial advantages observed with customized healing abutments are sustained over time. Further studies with extended follow-up periods are needed to determine the long-term stability and clinical relevance of these early outcomes. This study did not utilize cone-beam computed tomography (CBCT)-based planning or guided surgical protocols. These advanced techniques may influence clinical outcomes and could potentially skew the results.

## Conclusions

Within the limits of the study, the following conclusions were derived:

Papillary index scores were significantly higher for the cases done using custom-made healing abutments in both mesial and distal aspects when compared with cases using conventional healing abutments at the time of insertion. But there was no significant difference in the papillary scores between the custom-made healing abutment group and the conventional healing abutment group six months after insertion. Papillary index scores showed significant improvement with time in both groups within the observation period.

The custom-made healing abutment group had significantly higher PES than the conventional healing abutment group at the time of insertion and also after six months.

The study is unique in that it compares the effect of customized healing abutment on gingival aesthetics and papillary morphology with that of conventional healing abutment in a delayed implant loading protocol. This study concludes that customized healing abutments help in procuring a better emergence profile and aesthetics of the restoration. It also restores the optimum contour and papillary size at a faster rate than the conventional healing abutment in delayed loading implant cases.

.
